# PD-L1 Expression and Tumor Microenvironment Dynamics in Diffuse Large B-Cell Lymphoma: Immunophenotypic Insights

**DOI:** 10.25122/jml-2025-0078

**Published:** 2025-05

**Authors:** Georgian Halcu, Filip Cristian Mureșan, Andrei Niculae, Anca Evsei-Seceleanu, Mihai-Emilian Lapadat, Mihai Adrian Cerbu, Mihail Ceausu

**Affiliations:** 1 Department of Pathology, Carol Davila University of Medicine and Pharmacy, Bucharest, Romania; 2 Department of Pathology, Coltea Clinical Hospital, Bucharest, Romania; 3 Department of Pathology, Bucharest Emergency University Hospital, Bucharest, Romania; 4 Department of Pathology, Victor Babes National Institute for Research and Development in Pathology and Biomedical Sciences, Bucharest, Romania; 5 Department of Pathology, Sf. Maria Clinical Hospital, Bucharest, Romania; 6 Department of Pathology, C. I. Parhon National Institute of Endocrinology, Bucharest, Romania

**Keywords:** B-cell lymphoma, PD-L1, tumor microenvironment, immunohistochemistry

## Abstract

Diffuse large B-cell lymphoma (DLBCL) is the most common subtype of B-cell non-Hodgkin lymphoma (NHL) and is characterized by significant biological and clinical heterogeneity. The programmed death 1 (PD-1)/ programmed death-ligand 1 (PD-L1) immune checkpoint pathway plays a crucial role in tumor immune evasion; however, its diagnostic and prognostic relevance in DLBCL remains unclear. We retrospectively analyzed 66 cases of DLBCL diagnosed between 2017 and 2024 at a single institution. Immunohistochemistry was performed on formalin-fixed, paraffin-embedded tissues using antibodies against PD-L1, PD-1, CD4, CD8, and CD68. Clinical data and histopathological features were correlated with marker expression. Statistical analyses were conducted using IBM SPSS 25, with a significance level set at *P* < 0.05. PD-L1 expression (greater than 1%) in tumor cells was infrequent (6/66 cases), while immune cell PD-L1 positivity was prevalent (39/66 cases). PD-1 positivity was observed in five tumor samples and 40.9% of stromal immune cells. A significant association was found between tumoral PD-L1 expression and histological subtype (*P* = 0.015), with anaplastic variants showing higher expression levels. Positive PD-1 expression in immune cells was significantly associated with female gender (*P* = 0.044). High CD68 expression correlated with a lower Ann Arbor stage (*P* = 0.040) and tumor morphology (*P* = 0.010). CD4 and CD8 expression levels showed no significant correlations with clinicopathological features. PD-L1 and PD-1 expression patterns in DLBCL highlight their potential relevance for immune evasion and prognosis, particularly in anaplastic variants. The tumor microenvironment, especially macrophage infiltration, plays a complex role in disease progression. Further studies are needed to validate these findings and investigate therapeutic implications.

## INTRODUCTION

B-cell non-Hodgkin lymphomas (NHL) represent a diverse array of blood cancers, making up almost 4% of new cancer cases and cancer-related fatalities [1]. The group of large B-cell lymphomas encompasses a variety of tumours with different morphological, genetic, and clinical characteristics. Diffuse large B-cell lymphoma (DLBCL) NOS is the predominant subtype, characterized by its morphological features and mature B-cell phenotype [2,3]. Most cases of DLBCL-NOS broadly recapitulate the differentiation and maturation mechanisms active in normal B-cell development [4].

The programmed death-1 (PD-1)/programmed death-ligand 1 (PD-L1) pathway is an essential checkpoint for regulating T-cell–mediated immune responses. It comprises the transmembrane protein PD1/CD279 and its two ligands, PDL1 (CD274) and PDL2 (CD273). These PDLs activate PD1, leading to a reversible inhibition of T-cell activity and proliferation, which is also known as T-cell exhaustion or anergy. This mechanism plays an important physiological role in preventing placental infiltration by T cells and maintaining self-tolerance, which has been verified in animal studies of PD1 knock-out mice developing several autoimmune diseases [5].

Malignant tumors use the immunosuppressive PD-1/PD-L1 axis to avoid immune detection, as shown by increased PD-1 levels on tumor-infiltrating T cells [6,7]. The expression of PD-L1 by tumors further aids in immune evasion, a phenomenon seen in various solid tumors (such as lung and breast cancers) and hematolymphoid malignancies, including angioimmunoblastic T-cell lymphoma, follicular lymphoma (FL), DLBCL, primary mediastinal B-cell lymphoma (PMBCL), and classical Hodgkin lymphoma (cHL), especially the nodular sclerosis subtype.

In PMBCL and cHL, the recurrent amplifications of the PD-L1 gene locus at 9p24.1 highlight the importance of this pathway [8]. While PD-L1 expression has been identified in certain lymphomas through immunohistochemistry, research has often been constrained by small cohort sizes and inconsistency in antibody performance. Since cancer-specific T-cell responses might be suppressed through PD-1/PD-L1 interactions, blocking this pathway therapeutically has emerged as a promising approach, with early clinical trials showing positive results in cHL, melanoma, and non–small cell lung cancer [9].

The diagnostic and prognostic significance of PD-L1 expression through immunohistochemistry in lymphomas, especially in large patient groups, has not been definitively determined. In this study, we aimed to carefully examine PD-L1 expression in various DLBCL cases to explore its potential diagnostic value and relevance to clinicopathological features.

## MATERIAL AND METHODS

### Case selection

A retrospective study was designed at a single center, encompassing 66 cases of formalin-fixed, paraffin-embedded (FFPE) tissue specimens of DLBCL. The study followed the principles outlined in the Declaration of Helsinki (1975, revised in 2013). Following authorization from the Ethics and Research Committee of Coltea Clinical Hospital, adult patients (over 18 years old) who received diagnosis and treatment at the Department of Hematology, Coltea Clinical Hospital, Bucharest, Romania, were included in the study.

The inclusion criteria required a confirmed diagnosis of DLBCL, complete medical records, and available immunohistochemistry data. Exclusion criteria ruled out patients with specific large B-cell lymphomas, including primary CNS, cutaneous, intravascular, Burkitt, grey zone, plasmablastic, high-grade NOS, and primary mediastinal types. Individuals with HIV/AIDS, with inconsistent treatment for three years, or with significant treatment interruptions were also excluded, along with those having concurrent solid tumors or severe comorbidities.

The biopsy materials, consisting of either resection specimens or core needle samples, from patients diagnosed between 2017 and 2024, were thoroughly reviewed by pathologists (GH, MC, MB, and AE) to confirm the diagnoses according to the World Health Organization 5^th^ edition of the Classification of Hematolymphoid Tumors (WHO-HAEM5) guidelines.

Clinical data and all relevant information (histology/morphology, cell-of-origin/COO, bone marrow involvement, etc.) were retrieved from the electronic medical records between November 18, 2024, and December 31, 2024. The information was documented using a coding system to maintain participant confidentiality and identities. Initially, 125 patients were enrolled; however, half lacked adequate information in the hospital’s electronic system or opted to seek treatment elsewhere.

### Morphology and immunohistochemistry

Paraffin blocks were sectioned into 3-micrometre-thick sections and stained with standard hematoxylin and eosin (H&E) using routine laboratory procedures. The histopathological features of these lesions were assessed and recorded in our database.

Sections of 3 μm, procured from FFPE samples, were meticulously sectioned and affixed to positively charged microscope slides. Subsequently, these slides were heated at 60°C for 1 hour in a dry oven, thereby augmenting the tissue’s adhesion and facilitating the paraffin’s softening.

The immunohistochemistry process followed the established protocol, utilizing a Ventana BenchMark ULTRA autostainer. The sections were subjected to deparaffinization, rehydration, and antigen retrieval using Ventana’s CC1 solution (prediluted, pH 8.0). Primary antibodies were applied following the manufacturer’s dilution recommendations and allowed to interact with the sections.

The primary antibodies utilized in this study included the following: VENTANA PD-L1 (SP142, Roche, ready-to-use), PD-1 (NAT105) Mouse Monoclonal Antibody (Roche, ready-to-use), CONFIRM anti-CD4 (SP35) Rabbit Monoclonal Primary Antibody (Roche, ready-to-use), CONFIRM anti-CD8 (SP57) Rabbit Monoclonal Primary Antibody (Roche, ready-to-use), and CONFIRM anti-CD68 (KP-1) Primary Antibody (Roche, ready-to-use). Negative/positive controls were established as recommended.

The incubation periods were 16 minutes for PD-L1, 16 minutes for PD-1, 32 minutes for CD4, 20 minutes for CD8, and 4 minutes for CD68. The visualization process used the Optiview and the Ultraview universal DAB IHC detection kit as recommended, followed by counterstaining utilizing hematoxylin and a bluing solution. The slides were meticulously cleaned and dehydrated through a graded series of ethanol and xylene. Finally, the slides were affixed with mounting media onto microscope slides.

The Aperio GT 450 DX digital pathology slide scanner was used to evaluate the slides at the highest magnification (40x). The digital slides were evaluated using Aperio ImageScope Version 12.4.6.5003 (Leica Biosystems).

### Staining results

We stained non-small cell lung cancer and tonsil tissue to evaluate and assess the optimal staining for PD-L1. The pattern was as expected: membranous with occasional dot-like structures corresponding to the interaction sites.

PD-L1 was scored in the tumor and the immune cells as follows: <1%, 1-49%, and more than 50%, considering the entire available tumor across all cores for the individual cases.

Regarding PD-1, this marker was evaluated as negative or positive in both the tumor and immune cells (IC) in the background.

**Figure 1:**
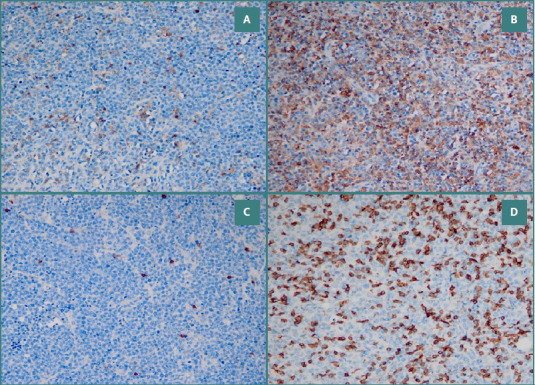
Tumor microenvironment in DLBCL A, CD4 low expression 20x; B, CD4 high 20x; C, CD8 low 20x; D, CD8 high 20x.

**Figure 2:**
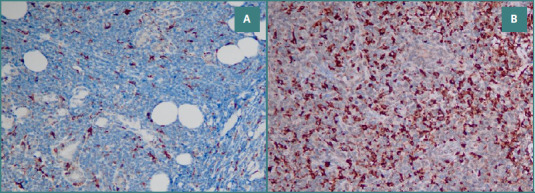
Tumor microenvironment in DLBCL. A, CD68 low expression 20x; B, CD68 high 20x

For CD4 (a marker for T helper cells), CD8 (a marker for T cytotoxic lymphocytes), and CD68 (a marker for macrophages/histiocytes), we counted positive stromal immune cells at 20x magnification in the three most abundant hotspots. We evaluated their expression as high or low (see Figure 1 and Figure 2).

### Statistical analysis

Data from the study was analyzed using IBM SPSS Statistics 25 and illustrated using Microsoft Office Excel/Word 2024. Qualitative variables were written as counts or percentages and were tested between groups using Fisher’s Exact Test/Pearson Chi-Square Test. Quantitative variables were written as medians with interquartile ranges. The threshold considered for the significance level for all tests was α = 0.05.

## RESULTS

Our study comprised 66 patients, whose key clinical characteristics are summarized in Table 1. The median age at diagnosis was 61.81 years (IQR = 49.14–73.83), with the majority of individuals being 60 years old or more; 59.1% of the patients were male. Upon revision, the Hans algorithm indicated that 65.2% of the cases were classified as the non-GCB subtype (ABC).

**Table 1:** Characteristics of the analysed patients

Parameter	Value
Age (Median [IQR])	61.81 (49.14-73.83)
Age ≥ 60 years (*n*, %)	36 (54.5%)
Sex (Male) (*n*, %)	39 (59.1%)
Histology (*n*, %)	
Centroblastic	43 (65.2%)
Immunoblastic	16 (24.2%)
Anaplastic	4 (6.1%)
High-grade	3 (4.5%)
Bone marrow involvement (*n*, %)	8 (12.3%)
Ann-Arbor stage (III-IV) (*n*, %)	55 (83.3%)
High LDH (Nr., %)	30 (45.5%)
IPI high risk score (≥3) (*n*, %)	43 (65.2%)
Cell of origin (Non-GCB) (*n*, %)	43 (65.2%)
PD-1 tumoral expression (*n*, %)	5 (7.6%)
PD-1 IC expression (*n*, %)	27 (40.9%)
PDL-1 tumour expression	<1% – 61 (92.4%), 1 to 49% – 4 (6.1%), >50% – 1 (1.5%)
PDL-1 IC expression	<1% – 27 (40.9%), 1 to 49% – 36(54.5%), >50% – 3 (4.5%)
CD68 high expression (*n*, %)	23 (34.8%)
CD4 high expression (*n*, %)	34 (51.5%)
CD8 high expression (*n*, %)	14 (21.2%)

Eight cases (12.3%) exhibited bone marrow involvement due to lymphoma. While most cases displayed a centroblastic morphology, four presented with anaplastic characteristics, and three demonstrated a high-grade appearance.

PD-L1 expression was observed as follows: one case (1.5%) exhibited positivity in greater than 50% of the tumor cells, while five cases demonstrated more than 1% expression in the tumor cells. Additionally, immune cells within the stroma with positivity in more than 1% were seen in 39 instances.

The ligand PD-1 was positive in the tumor cells of five cases, while it was 40.9% positive in the stromal immune cells.

High expression of CD68, CD4, and CD8 was noted in 23 cases, 34 cases, and 14 cases, respectively.

Table 2 delineates the distribution of patients according to PD-1 marker expression and other characteristics. The findings reveal that the majority of examined associations between the expression of tumour or immune cell (IC) PD-1 and the different parameters were not statistically significant (*P* > 0.05), except the relationship between gender and PD-1 expression in the immune cells (*P* = 0.044).

**Table 2:** Distribution of the patients according to PD-1 marker expression and other characteristics

PD-1 (*n*, %)	Tumoral		P*	IC		P*
	Negative	Positive		Negative	Positive	
**Age**						
< 60	27 (44.3)	3 (60)	0.652	14 (35.9)	16 (59.3)	0.080
≥ 60	34 (55.7)	2 (40)		25 (64.1)	11 (40.7)	
**Sex**						
Female	26 (42.6)	1 (20)	0.641	12 (30.8)	15 (55.6)	0.044**
Male	35 (57.4)	4 (80)		27 (69.2)	12 (44.4)	
**COO**						
GCB	21 (34.4)	2 (40)	1.000	13 (33.3)	10 (37)	0.797
Non-GCB	40 (65.6)	3 (60)		26 (66.7)	17 (63)	
**Bone marrow involvement**						
Absent	52 (86.7)	5 (100)	1.000	34 (87.2)	23 (88.5)	1.000
Present	8 (13.3)	0 (0)		5 (12.8)	3 (11.5)	
**Ann-Arbor**						
I-II	10 (16.4)	1 (20)	1.000	5 (12.8)	6 (22.2)	0.336
III-IV	51 (83.6)	4 (80)		34 (87.2)	21 (77.8)	
**LDH**						
Normal	33 (54.1)	3 (60)	1.000	22 (56.4)	14 (51.9)	0.803
High	28 (45.9)	2 (40)		17 (43.6)	13 (48.1)	
**IPI score**						
0-2	21 (34.4)	2 (40)	1.000	10 (25.6)	13 (48.1)	0.071
≥ 3	40 (65.6)	3 (60)		29 (74.4)	14 (51.9)	
**Histology**						
Centroblastic	41 (67.2)	2 (40)	0.239	25 (64.1)	18 (66.7)	0.929
Immunoblastic	14 (23)	2 (40)		9 (23.1)	7 (25.9)	
Anaplastic	3 (4.9)	1 (20)		3 (7.7)	1 (3.7)	
High-grade	3 (4.9)	0 (0)		2 (5.1)	1 (3.7)	

* Fisher’s Exact Test, **Pearson Chi-Square Test

In this case, female patients exhibited a notably higher association with positive expression of IC PD-1, with rates of 55.6% compared to 30.8% for their male counterparts.

Table 3 shows the patients’ distribution according to PDL-1 expression and other characteristics. Results show that most tested associations between expression of tumoral or immune cells, PDL-1, and the different parameters were not significant (*P* > 0.05), except for histology and tumoral PDL-1 (*P* = 0.015). According to Z-tests with Bonferroni correction, centroblastic lymphomas were more associated with negative expression (68.9% vs. 20%), while anaplastic ones were more related to positive expression (40% vs. 3.3%).

**Table 3:** Distribution of patients according to PDL-1 marker expression and other characteristics

PDL-1 (*n*, %)	Tumoral		P*	IC		P*
	< 1	≥ 1		< 1	≥ 1	
**Age**						
< 60	27 (44.3)	3 (60)	0.652	14 (51.9)	16 (41)	0.455
≥ 60	34 (55.7)	2 (40)		13 (48.1)	23 (59)	
**Sex**						
Female	27 (44.3)	0 (0)	0.073	10 (37)	17 (43.6)	0.621
Male	34 (55.7)	5 (100)		17 (63)	22 (56.4)	
**COO**						
GCB	22 (36.1)	1 (20)	0.651	7 (25.9)	16 (41)	0.294
Non-GCB	39 (63.9)	4 (80)		20 (74.1)	23 (59)	
**Bone marrow involvement**						
Absent	52 (86.7)	5 (100)	1.000	24 (88.9)	33 (86.8)	1.000
Present	8 (13.3)	0 (0)		3 (11.1)	5 (13.2)	
**Ann-Arbor**						
I-II	9 (14.8)	2 (40)	0.191	7 (25.9)	4 (10.3)	0.108
III-IV	52 (85.2)	3 (60)		20 (74.1)	35 (89.7)	
**LDH**						
Normal	34 (55.7)	2 (40)	0.652	15 (55.6)	21 (53.8)	1.000
High	27 (44.3)	3 (60)		12 (44.4)	18 (46.2)	
**IPI score**						
0-2	21 (34.4)	2 (40)	1.000	11 (40.7)	12 (30.8)	0.440
≥ 3	40 (65.6)	3 (60)		16 (59.3)	27 (69.2)	
**Histology**						
Centroblastic	**42 (68.9)**	**1 (20)**	**0.015**	21 (77.8)	22 (56.4)	0.115
Immunoblastic	14 (23)	2 (40)		3 (11.1)	13 (33.3)	
Anaplastic	**2 (3.3)**	**2 (40)**		1 (3.7)	3 (7.7)	
High-grade	3 (4.9)	0 (0)		2 (7.4)	1 (2.6)	

* Fisher’s Exact Test

Data from Table 4 shows the distribution of patients according to the expression of different markers and other characteristics. The results indicate that most tested associations between the expression of CD68, CD8, and CD4 and other parameters were not significant (*P* > 0.05), except for the Ann Arbour stage and CD68 (*P* = 0.040), where patients with stage III-IV were significantly less associated with high CD68 (30.4% vs. 9.3%). For histology and CD68 (*P* = 0.010), according to Z-tests with Bonferroni correction, centroblastic tumours were associated with low expression (76.7% vs. 43.5%); in comparison, high-grade tumours exhibited a greater association with high expression (13% vs. 0%).

**Table 4:** Distribution of patients according to other marker expression and characteristics

Marker (*n*, %)	CD68		P*	CD8		P*	CD4		P*
	Low	High		Low	High		Low	High	
**Age**									
< 60	19 (44.2)	11 (47.8)	0.801	17 (53.1)	13 (38.2)	0.323	23 (44.2)	7 (50)	0.768
≥ 60	24 (55.8)	12 (52.2)		15 (46.9)	21 (61.8)		29 (55.8)	7 (50)	
**Sex**									
Female	18 (41.9)	9 (39.1)	1.000	16 (50)	11 (32.4)	0.211	21 (40.4)	6 (42.9)	1.000
Male	25 (58.1)	14 (60.9)		16 (50)	23 (67.6)		31 (59.6)	8 (57.1)	
**COO**									
GCB	15 (34.9)	8 (34.8)	1.000	11 (34.4)	12 (35.3)	1.000	17 (32.7)	6 (42.9)	0.536
Non-GCB	28 (65.1)	15 (65.2)		21 (65.6)	22 (64.7)		35 (67.3)	8 (57.1)	
**Bone marrow involvement**									
Absent	35 (83.3)	22 (95.7)	0.242	28 (87.5)	29 (87.9)	1.000	46 (88.5)	11 (84.6)	0.655
Present	7 (16.7)	1 (4.3)		4 (12.5)	4 (12.1)		6 (11.5)	2 (15.4)	
**Ann-Arbor**									
I-II	4 (9.3)	7 (30.4)	**0.040**	5 (15.6)	6 (17.6)	1.000	7 (13.5)	4 (28.6)	0.227
III-IV	39 (90.7)	16 (69.6)		27 (84.4)	28 (82.4)		45 (86.5)	10 (71.4)	
**LDH**									
Normal	23 (53.5)	13 (56.5)	1.000	19 (59.4)	17 (50)	0.470	26 (50)	10 (71.4)	0.228
High	20 (46.5)	10 (43.5)		13 (40.6)	17 (50)		26 (50)	4 (28.6)	
**IPI score**									
0-2	13 (30.2)	10 (43.5)	0.295	14 (43.8)	9 (26.5)	0.197	16 (30.8)	7 (50)	0.215
≥ 3	30 (69.8)	13 (56.5)		18 (56.2)	25 (73.5)		36 (69.2)	7 (50)	
**Histology**									
Centroblastic	**33 (76.7)**	**10 (43.5)**	**0.010**	23 (71.9)	20 (58.8)	0.430	36 (69.2)	7 (50)	0.398
Immunoblastic	8 (18.6)	8 (34.8)		5 (15.6)	11 (32.4)		11 (21.2)	5 (35.7)	
Anaplastic	2 (4.7)	2 (8.7)		2 (6.3)	2 (5.9)		3 (5.8)	1 (7.1)	
High-grade	**0 (0)**	**3 (13)**		2 96.3)	1 (2.9)		2 (3.8)	1 (7.1)	

* Fisher’s Exact Test

## DISCUSSION

This study examined how PD-L1 is expressed in lymphoma and infiltrating or surrounding immune cells. We used an antibody that provides the clearest signal for staining, ensuring our results are as reliable as possible. PD-L1 expression was observed: one case (1.5%) exhibited positivity in more than 50% of the tumor cells, while five cases demonstrated more than 1% expression in the tumor cells. Additionally, immune cells within the stroma with positivity in more than 1% were seen in 39 cases.

Our results show that most associations between PD-L1 expression and evaluated parameters were insignificant, except for morphological type and tumoral PD-L1 (*P* = 0.015). Centroblastic lymphomas were linked to negative expression, while anaplastic lymphomas were associated with positive expression. The relationship between PD-L1 expression and the anaplastic variant of DLBCL is of increasing interest in clinical and research settings, particularly due to the relevance of PD-L1 as a target for immunotherapy.

The expression of PD-L1 in diffuse large B-cell lymphoma bears substantial implications for patient prognosis and therapeutic responses. Recent studies indicate that the expression of PD-L1 correlates with diminished overall survival rates in patients diagnosed with DLBCL, especially in instances exhibiting chromosomal alterations at 9p24.1, where the PD-L1 gene is located [10]. Research indicates that activated B-cell-like (ABC) DLBCL typically exhibits greater PD-L1 levels than germinal center B-cell-like (GCB) types, linking PD-L1 expression to unfavorable prognostic outcomes [11,12].

Additionally, the tumor microenvironment (TME) is vital, with macrophages identified as key contributors to PD-L1 expression, highlighting the intricate relationships between tumor cells and immune system cells [13]. Structural differences affecting PD-L1 are often seen in Epstein-Barr virus (EBV)-related lymphomas, suggesting unique regulatory pathways for PD-L1 in these situations [14].

Interestingly, research indicates that changes in PD-L1 expression do not consistently reflect chromosomal amplification or mutations. In some instances, elevated PD-L1 levels are observed without any DNA modifications, indicating the presence of alternative regulatory mechanisms [15,16]. Consequently, although PD-L1 continues to represent a promising target for therapeutic intervention, the variability of its expression in DLBCL underscores the necessity for personalized treatment strategies that specifically concentrate on this pathway.

The ligand PD-1 was positive in the tumor cells of five cases, while the immune stromal cells were positive in 40,9% of cases. The analysis revealed that most associations between PD-1 expression in tumor or immune cells and the evaluated parameters were not statistically significant (*P* > 0.05), except for the association between sex and IC PD-1 expression (*P* = 0.044). Specifically, positive IC PD-1 expression was more frequent in female patients (55.6%) than in male patients (30.8%).

The expression of PD-1 in diffuse large B-cell lymphoma and its tumor microenvironment is critical for understanding immune evasion and therapeutic avenues. PD-1 is mainly found on tumor-infiltrating immune cells, and its interaction with PD-L1 effectively suppresses T-cell activation and proliferation, aiding tumor growth immunotolerance [17]. Research demonstrates that the aberrant expression of PD-1 and other immune checkpoints, including TIM-3 and LAG-3, is prevalent within the microenvironment of DLBCL, particularly associated with aggressive disease behavior [18].

Additional analyses indicate that PD-1 expression is linked with other immune checkpoint molecules, strengthening the tumor’s immune evasion tactics [19]. Notably, the tumor microenvironment in DLBCL contains multiple non-malignant cells, such as macrophages, that can also express PD-L1. This adds complexity to the immune regulatory network and enhances T-cell inhibition through PD-1/PD-L1 interactions [12,13]. This complexity highlights the necessity of examining the diverse role of TME in influencing tumor progression and reactions to new therapeutic strategies aimed at the PD-1/PD-L1 axis.

Understanding the dynamics of PD-1 in DLBCL and its interactions with cellular elements and immune checkpoint molecules in the TME is essential for developing more effective treatment strategies and improving patient prognostication.

Regarding the tumoral microenvironment in DLBCL, we stained our samples with CD68, CD4, and CD8. We evaluated the expression as high or low in the macrophages and lymphocytes in the tumor or the background. Our results showed high CD68, CD4, and CD8 expression in 23 cases, 34 cases, and 14 cases, respectively.

Statistical analysis indicated that most associations between the expression of CD68, CD8, and CD4 and other parameters were not significant (*P* > 0.05), except for the Ann Arbour stage and CD68 (*P* = 0.040), where patients with advanced were significantly less associated with high CD68 (30.4% vs. 9.3%).

Correlating morphology and CD68 expression (*P* = 0.010), according to Z-tests with Bonferroni correction, centroblastic tumours were associated with low expression of CD68 macrophages (76.7% vs. 43.5%); in comparison, high-grade tumours exhibited a greater association with high CD68 expression (13% vs. 0%).

The presence of CD68, a marker for pan-macrophages, in the TME of DLBCL offers valuable insights into the function of tumor-associated macrophages (TAMs) in tumor biology. CD68 is linked to M1 macrophages, which typically exhibit anti-tumoral properties, and M2 macrophages, which are recognized for their immunosuppressive traits [20]. Increased CD68+ macrophages within the TME have been associated with unfavorable prognoses in DLBCL, suggesting a potentially detrimental role in tumor progression [21,22].

A study by Serna *et al*. showed a predominant presence of CD68+ macrophages in DLBCL, correlating positively with pro-inflammatory markers, suggesting a complex relationship between inflammation and macrophage activity [20]. Marinaccio *et al*. noted that while CD68 marks TAMs, it is limited in accurately reflecting specific macrophage subtypes. High CD68 expression in TAMs generally indicates an adverse prognosis in various lymphomas [23].

Additionally, Hanamura *et al*. noted that although PD-L1 is found on some CD68+ macrophages, the general inflammatory environment created by these macrophages might aid tumor survival, highlighting their dual function in immune suppression and tumor support [24]. This dual nature complicates the landscape of macrophage involvement in DLBCL. For instance, CD68+ TAMs have been associated with therapy resistance and promote immunosuppressive environments that favor tumor growth [25].

Interestingly, the prognostic value of CD68+ macrophage infiltration is still debated. Certain studies indicate that elevated levels of CD68+ macrophages are linked to better patient outcomes when effectively treated with therapies like rituximab [21], while others point towards a poor prognosis associated with high TAM density in various lymphoma types [22]. This inconsistency highlights the necessity for additional research to elucidate the specific roles and impacts of CD68+ macrophages in DLBCL and their possible use as therapeutic targets.

CD68 is an essential indicator for evaluating macrophage presence in the tumor microenvironment of DLBCL. Its expression signifies the intricate interactions within the immune landscape of DLBCL, affecting clinical outcomes and treatment responses. Therefore, a detailed interpretation is crucial for its application in prognostic evaluations.

Understanding the expression and role of CD4+ and CD8+ T cells in the TME of DLBCL is essential for grasping the immune landscape and potential therapeutic approaches. DLBCL is marked by changes in T cell populations, notably demonstrating higher infiltration of CD8+ T cells relative to other hematologic malignancies, whereas the CD4+ T cell population is often considerably lower [26]. This imbalance may impact the overall immune response and prognosis.

Research by Wang *et al*. indicates that the percentage of CD8+ T cells increases in patients undergoing specific treatment regimens, highlighting the importance of these cells in anti-tumor immunity [27]. CD8+ T cells are predominantly cytotoxic and play a crucial role in eliminating tumor cells; however, their functionality can be compromised by immune checkpoint molecules, such as PD-1, which is frequently upregulated in DLBCL [26,28]. Moreover, CD4+ T cells can exhibit dual roles: they may support the activation of CD8+ T cells and help orchestrate the immune response. However, depending on their subtype and activation status, they can also engage in immunosuppressive activities [29,30].

In addition to the quantitative differences in CD4+ and CD8+ T cell populations, qualitative aspects are also important. PD-1 and other co-inhibitory molecules are commonly noted on CD4+ and CD8+ T cells in DLBCL, reflecting an exhausted T cell phenotype that may compromise effective immune responses [26,31].

The dynamics of CD4+ and CD8+ T cell presence and their functional status in the TME of DLBCL are critical for understanding tumor immunology and refining treatment approaches. The immune landscape is characterized by an increased presence of CD8+ T cells, decreased populations of CD4+ T cells, and significant expression of immune checkpoints that may inhibit their effective anti-tumor activity.

In conclusion, our study shows the diverse PD-L1 and PD-1 expression in tumor cells and the immune microenvironment of DLBCL. We found significant links between morphological types, especially connecting anaplastic lymphomas to higher tumoral PD-L1 levels, suggesting responsiveness to PD-1/PD-L1 immunotherapy. Additionally, PD-1 positivity in immune cells was more common in female patients, indicating potential sex-related immunological differences needing further investigation.

## CONCLUSION

Our investigation into the tumor microenvironment highlights the complex roles of macrophages (CD68+) and T-cells (CD4+, CD8+). High macrophage infiltration correlated with advanced Ann Arbor stages and high-grade morphologies, demonstrating their dual role in tumor progression and immunosuppression. While CD4+ and CD8+ T-cell levels were not significantly correlated with clinical parameters, their presence and immune checkpoint expression emphasize their critical role in shaping DLBCL’s immunological landscape.

These findings reinforce the importance of immunophenotypic profiling in DLBCL, highlighting PD-L1, PD-1, and immune cell infiltration as potential prognostic and treatment biomarkers. Further research is needed to clarify the mechanisms of immune modulation, enabling personalized therapies and improving patient outcomes.
